# Climate change, women’s health, and the role of obstetricians and gynecologists in leadership

**DOI:** 10.1002/ijgo.13958

**Published:** 2021-10-25

**Authors:** Linda C. Giudice, Erlidia F. Llamas‐Clark, Nathaniel DeNicola, Santosh Pandipati, Marya G. Zlatnik, Ditas Cristina D. Decena, Tracey J. Woodruff, Jeanne A. Conry, the FIGO Committee on Climate Change and Toxic Environmental Exposures

**Affiliations:** ^1^ Department of Obstetrics, Gynecology and Reproductive Sciences University of California, San Francisco San Francisco California USA; ^2^ Department of Obstetrics and Gynecology Division of Ultrasound University of the Philippines ‐ Philippine General Hospital Manila Philippines; ^3^ Department of Obstetrics and Gynecology Johns Hopkins Health System Washington District of Columbia USA; ^4^ Obstetrix Medical Group/Mednax (Maternal‐Fetal Medicine) Campbell California USA; ^5^ Department of Obstetrics, Gynecology and Reproductive Sciences Division of Maternal Fetal Medicine Program on Reproductive Health and the Environment Environmental Research and Translation for Health (EaRTH) Center University of California, San Francisco San Francisco California USA; ^6^ Departments of Anatomy Clinical Epidemiology and Public Health International University of Santo Tomas Manila Philippines; ^7^ Program on Reproductive Health and the Environment Environmental Research and Translation for Health (EaRTH) Center University of California, San Francisco San Francisco California USA; ^8^ FIGO London UK

**Keywords:** advocacy, climate change, education, environment, reproduction, women's health

## Abstract

Climate change is one of the major global health threats to the world's population. It is brought on by global warming due in large part to increasing levels of greenhouse gases resulting from human activity, including burning fossil fuels (carbon dioxide), animal husbandry (methane from manure), industry emissions (ozone, nitrogen oxides, sulfur dioxide), vehicle/factory exhaust, and chlorofluorocarbon aerosols that trap extra heat in the earth's atmosphere. Resulting extremes of weather give rise to wildfires, air pollution, changes in ecology, and floods. These in turn result in displacement of populations, family disruption, violence, and major impacts on water quality and availability, food security, public health and economic infrastructures, and limited abilities for civil society to maintain citizen safety. Climate change also has direct impacts on human health and well‐being. Particularly vulnerable populations are affected, including women, pregnant women, children, the disabled, and the elderly, who comprise the majority of the poor globally. Additionally, the effects of climate change disproportionally affect disadvantaged communities, including low income and communities of color, and lower‐income countries that are at highest risk of adverse impacts when disasters occur due to inequitable distribution of resources and their socioeconomic status. The climate crisis is tilting the risk balance unfavorably for women's sexual and reproductive health and rights as well as newborn and child health. Obstetrician/gynecologists have the unique opportunity to raise awareness, educate, and advocate for mitigation strategies to reverse climate change affecting our patients and their families. This article puts climate change in the context of women's reproductive health as a public health issue, a social justice issue, a human rights issue, an economic issue, a political issue, and a gender issue that needs our attention now for the health and well‐being of this and future generations. FIGO joins a broad coalition of international researchers and the medical community in stating that the current climate crisis presents an imminent health risk to pregnant people, developing fetuses, and reproductive health, and recognizing that we need society‐wide solutions, government policies, and global cooperation to address and reduce contributors, including fossil fuel production, to climate change.

## Synopsis

The climate crisis is impacting air quality, food security, and women's sexual and reproductive health and rights. Obstetrician/gynecologists can work to help mitigate its consequences.

## 1 INTRODUCTION

Investing in the health of women is investing in the health of current and future generations. Whether we as obstetrician/gynecologists (OBGYNs) are providing care for individual patients, guiding women's health services, leading global efforts to reduce maternal morbidity and mortality, reducing cervical cancer, directing global fistula programs, championing reproductive health access, eliminating exposures to toxic chemicals, or being a voice in the fight against climate change, we are uniquely qualified to advocate, teach, and provide research on behalf of women. The work of FIGO (the International Federation of Gynecology and Obstetrics) is built on four pillars: advocacy, research interpretation, capacity building, and education. FIGO joins a broad coalition of international researchers and the medical community in stating that the current climate crisis presents an imminent health risk to pregnant people, developing fetuses, and reproductive health. Our goal broadly is to summarize the research specific to women's health and help OBGYNs amplify their roles through education, research, and advocacy. In particular, we join other organizations in recognizing that we need society‐wide solutions, government policies, and global cooperation to address and reduce contributors, including fossil fuel production, to climate change. This review focuses on the climate crisis and women's reproductive health.

## 2 THE SCOPE OF THE PROBLEM

Year over year, record‐setting data are observed for mean global temperature, rainfall, fires, storms, and vector‐borne illnesses.^1^ The common denominator is the impact of climate change, accompanied by impacts on health, the effects of which (e.g. air pollution) are also projected to exacerbate the effects of the coronavirus disease 2019 (COVID‐19) pandemic.^2^ The *Lancet Countdowns* on health and climate change in 2019 and 2020 convened 35 academic institutions and United Nations’ agencies.^1^, ^3^ Their 2019 summary underscored unprecedented population exposures to wildfires, 475 million additional heat wave events globally in 2019, and global warming projected to exceed 3°C increase in average temperature by the end of this century, deemed by the United Nations’ Intergovernmental Program for Climate Change as “code red” for the planet.^4^ These conditions are ideal for expansion of vector‐borne illnesses and derive from changes in frequency, duration, and severity of weather resulting in droughts, heavy rains, rising sea levels and waterways, high temperatures, and storms, including hurricanes, cyclones, and snowstorms. The World Health Organization (WHO) has reported that natural disasters have tripled since 1960, translating into more than 60 000 deaths annually.^5^ WHO forecasts that by 2030 this effect will increase to at least 250 000 deaths annually. It is the impact on the air we breathe, the water we drink, the food we consume, and our ability to seek shelter that threatens global health; as OBGYNs, we understand that these factors directly impact reproductive health and women's choices. There is a host of effects from disasters, including direct impacts on infrastructure, homes, and farms, and indirect effects on health that can be serious and long standing, even affecting future generations. Moreover, death rates from disasters are four‐fold higher in under‐resourced countries.^6^

The impacts of climate change threaten daily life and survival: from food and shelter insecurity, to decreased agricultural production and vector‐borne illnesses. The 2020 *Lancet Countdown* on health and climate change summarized 43 key indicators across five domains ranging from impacts and vulnerabilities to economics and political engagement.^3^ The stability of communities and health systems is at stake, with higher vulnerabilities in under‐resourced countries. Climate change effects are not uniformly distributed across the globe, and populations, including children, women, pregnant women, the elderly, marginalized peoples, and refugees are impacted disproportionately.^3^ In 2002, Cannon et al.^7^ underscored gender disparities and climate change in Bangladesh, where women are more vulnerable than those in highly resourced countries because of socially determined roles, lack of food and shelter, and lack of access to hygiene. Seventy percent of the 1.3 billion people in low‐resource countries living below the poverty level are women, and climate change places this group in the most vulnerable position.^8^ Indeed, women are adversely affected more than men worldwide, and it is anticipated that without proper adaptation, this disparity will only worsen in the coming decades.

It is incumbent upon OBGYNs to understand how climate change impacts the women we serve and advocate with them and on their behalf. Thus, it is essential that, as healthcare providers to women, we need to understand the scientific basis underlying the effects of climate change on women's health outcomes. The purpose of this article is to summarize the evidence for climate change effects on women's health and to recommend strategies to mitigate these effects through education, advocacy, and collaboration. In particular, we note that while individuals have some control over mitigating the harms of climate change, they are unable to address the root causes and thus we advocate for governments across the globe to implement policies that address and reduce the root causes of climate change. We conclude with a case study of an action plan in the Philippines to address strategies for change and resilience.

## 3 CLIMATE CHANGE AND REPRODUCTIVE HEALTH OUTCOMES

**FIGURE 1:**
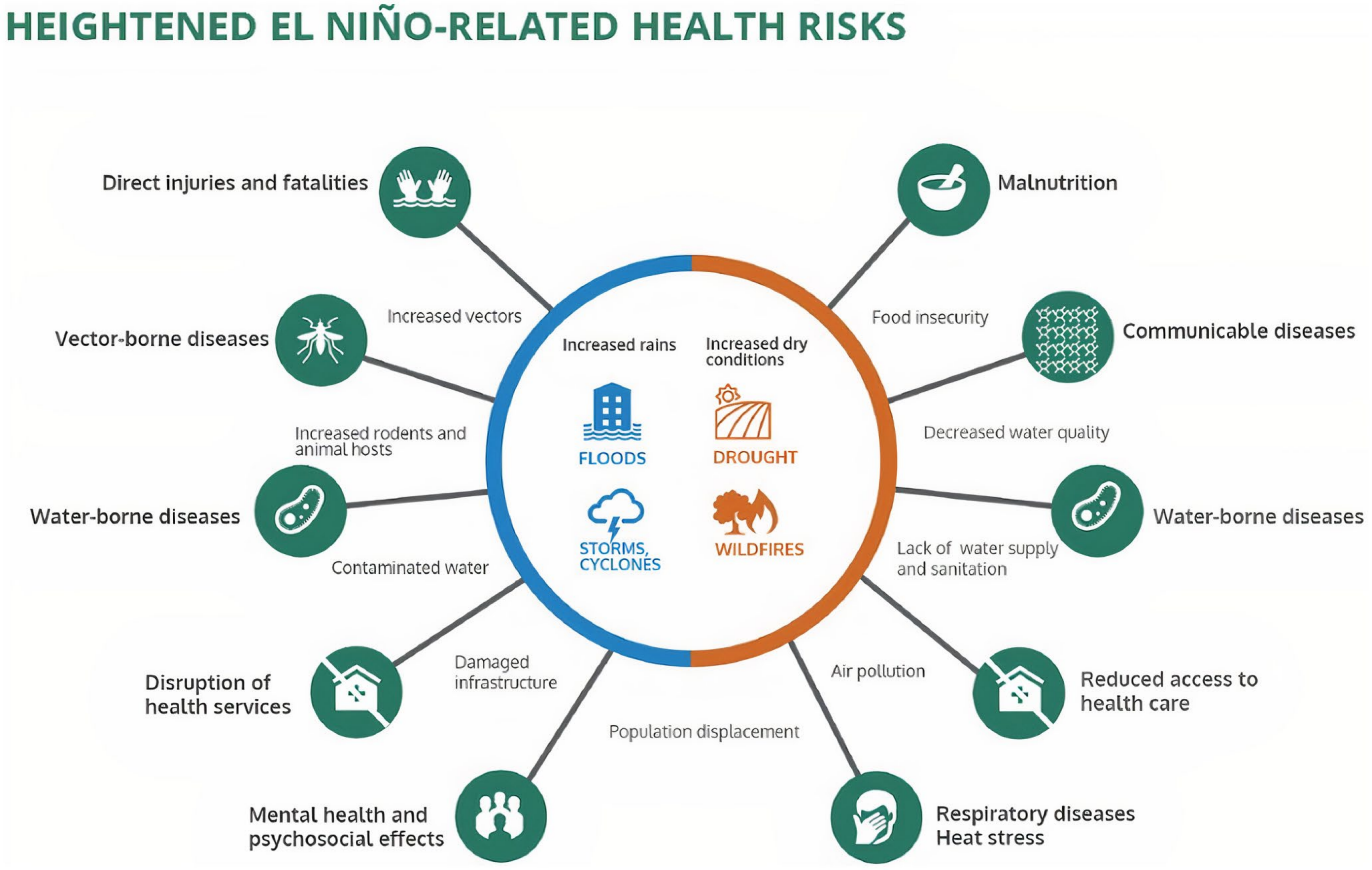
Health consequence of extreme climate‐related events. Source: WHO 2016. Global report on El Niño and health: https://www.who.int/news‐room/feature‐stories/detail/el‐ni%C3%B1o‐affects‐more‐than‐60‐million‐people. Accessed September 27, 2021

Health consequences of extreme climate‐related events are classified as direct or indirect.^9^ Direct effects relate to the physiologic impacts of heat or cold and the cellular and organismal responses to pollution, water contaminants, or disruption of services. Indirect impacts relate to vectors and pathogens, whose increases or distribution are ultimately a result of climate change. Migration and civil conflict may be the result of both direct and indirect climate events (Figure 1). Epidemiologic evidence supports the direct impact of climate change on fertility, prenatal outcomes, mental health, sexual health and reproductive rights, and survival.^10^, ^11^ Depending on the disaster, communities have experienced post‐traumatic stress, suicides, and adverse pregnancy outcomes in those who survive.^12^ Likewise, epidemiologic research has shown the susceptibility of women to these indirect shifts in vector prevalence and distribution. Within under‐resourced countries, access to adequate health care, including reproductive health needs like contraceptives and abortions, or pre‐pregnancy, prenatal, and maternity health care is already lacking, and any disaster that limits access will further exacerbate outcomes. In a systematic review, Hartville et al.^13^ found that disasters have the potential to impact maternal mental health and perinatal outcomes. We address several specific concerns: air pollution, heat, the synergistic effects of the two, and flooding in the sections that follow.

### 3.1 Air pollution

Climate change and air pollution are tightly interwoven. Fossil fuel consumption drives climate change and air pollution. Carbon dioxide, methane, and nitrous oxide—the most common by‐products of such consumption—are the major contributors to climate change. Such air pollutants affect incoming sunlight that is reflected or absorbed by the atmosphere, thus exacerbating climate change. Air pollutants are airborne solids, liquids, or gases that can impact health. The major air pollutants include ozone, carbon monoxide, sulfur dioxide, and particulate matter less than 2.5 and 10 microns (PM_2.5_ and PM_10,_ respectively).^14^ Air pollution can damage vital organs, including the lungs, heart, and placenta. Global deaths attributed to fine particulate matter (PM_2.5_) from fossil fuel combustion were estimated at 10.2 million in 2012, with 62% in China (3.9 million) and India (2.5 million).^15^ Air pollution in the Ukraine has been linked to 21% of all illnesses affecting women and children.^16^ Air pollution has been linked to hypertensive disorders in pregnant women and other vulnerable populations.^17^

Numerous studies have demonstrated a link between prepregnancy and prenatal exposure to air pollutants and lower fertility and live birth rates in spontaneous conceptions and after in vitro fertilization and embryo transfer (IVF‐ET),^18^, ^19^, ^20^ and adverse obstetric outcomes with higher rates of miscarriage,^21^, ^22^ preterm birth,^23^, ^24^, ^25^, ^26^, ^27^ and low birthweight.^24^, ^25^, ^26^, ^27^ These articles include systematic reviews with meta‐analysis: level 1 evidence according to the US Agency for Healthcare Research and Quality.^18^, ^26^, ^27^ Residential proximity to major roadways increased risk of infertility among nurses in the USA^19^ and couples undergoing IVF.^20^ Among the latter, the adjusted percentage of IVF cycles resulting in live birth was 33% for those living less than 50 m from a major roadway versus 46% for more than 400 m from a major roadway. A 2016 meta‐analysis found that over 3% of preterm birth in the USA could be attributed to PM_2.5_,^28^ and a 2017 meta‐analysis found that globally PM_2.5_‐associated preterm birth was as high as 18%, with highest risk in South Asia, East Asia, North Africa/Middle East, and West Sub‐Saharan Africa.^29^ Research has also demonstrated a proximity effect for prenatal pollutant exposures and low birthweight: a 2017 study found that among 1.1 million live births, the risk of low birthweight was higher within 3 km of a fracking site compared to the background risk and increased by 25% within 1 km of a site.^30^ Furthermore, international collaborations found air pollution linked to decreases in birthweight.^31^, ^32^ In California, closing eight coal and oil power plants reduced air pollution over a 10‐year period and was accompanied by a reduction in preterm births that could not be explained by any other causes.^33^ With wildfires and firestorms increasing as a result of global warming, there is a significant seasonal impact on air quality and thus the risk of perinatal complications. It is the impact of both temporal and spatial exposure to fire and the concomitant pollution that needs further study. Mendola et al.^34^ proposed that regional changes in air pollution can have impacts on consecutive pregnancies, with increased exposure to pollutants having higher risk of adverse outcomes. Recent research based on California wildfires confirms that exposure to wildfire smoke during pregnancy is associated with preterm birth and moreover suggests an adaptive response to prior exposure.^35^ Specifically, mothers in regions with infrequent smoke exposure had substantially greater impacts from an additional smoke day than those in regions where smoke exposure was more common.^35^

### 3.2 Heat

In just over a century, climate change has warmed the average earth temperature by 1.2°C, and many parts of the globe have warmed up to 3°C.^4^ These extremes in temperature have resulted in stresses to ecosystems and life. In the USA, extreme heat causes more fatalities than any other weather hazard.^36^ Even a small change in average temperature results in marked increases in temperature extremes. Witness the Mediterranean, where heat extremes are predicted to increase by 200%–500%.^37^ The seven warmest years on record have been the last seven years (2015 onward) with 2020 tied for the warmest ever.^38^ Such temperature extremes have the potential to decrease crop production and increase food scarcity,^39^ increase water shortages and drought, and increase human exposures to heat‐related illness^40^ at work and at home. Early reports on the June 2021 heat wave in the Pacific Northwest suggest more than 600 excess deaths in Oregon and Washington states.^41^

In addition to the numerous sequelae of extreme weather and natural disasters on maternal and child health^42^—including food insecurity, water contamination, increased risk of vector‐borne illness, mental trauma due to displacement and violence against women^43^—specific obstetric risks linked to heat include preterm birth and low birthweight. Five international review articles found heat to be associated with preterm birth,^44^, ^45^, ^46^, ^47^, ^48^ corroborating findings from multiple national studies.^49^, ^50^ Additionally, four of these international reviews found heat to be associated with low birthweight,^44^, ^46^, ^48^, ^50^ which are consistent with data from the USA.^50^, ^51^ A scoping review identified that the overwhelming majority of peer‐reviewed publications on this subject identified a strong positive correlation between heat and air pollution exposure during pregnancy with increased risks of stillbirth, low birthweight, and preterm birth.^27^ No critical period of maternal sensitivity to heat has yet been definitively identified, but data are suggestive that heat exposure earlier in a warm season is more harmful than later due to lack of acclimatization.^47^

In addition to prematurity, low birthweight, and stillbirth, in utero heat exposure has also been attributed to significant increase in risk for fetal congenital anomalies, including conotruncal and septal heart defects^52^, ^53^ and cataracts.^54^ Some areas of the USA are anticipated to have up to a 60% increase in incidence of fetal congenital heart defects by 2035 due to heat exposure. Prenatal heat exposure has been linked to decreased cognitive ability and diminished adult earnings.^55^, ^56^

Maternal health is also at risk with heat exposure, with studies identifying increased incidence of maternal hypertensive disease^57^ and placental abruption.^58^

Extreme heat with concomitant pest damage and water shortages potentially lead to decreased crop production.^59^ Food insecurity adversely impacts women and children, increases likelihood and magnitude of population migrations, and places women at risk for mental and physical stress.

### 3.3 Synergistic effects: air pollution and heat

Emerging evidence suggests that in addition to being independent risk factors for adverse fertility and obstetric outcomes, air pollution and heat can combine to create synergistic risk. One representative study from Massachusetts in the USA found that 5 μg/m^3^ increase in average third trimester PM_2.5_ exposure was linked to point estimates of 1.7–10 gestational age reduction across the gestational age percentiles for subpopulations with high average ambient temperature (≥21°C) in the third trimester.^60^ Similar risk was also found for a subpopulation with low average ambient temperature (≤−0.59°C) in the third trimester,^60^ highlighting that both extremes of temperature may be cause for concern. Several other studies conducted in Asia have corroborated this newly identified potential for synergistic risk.^61^, ^62^

### 3.4 Floods

Flooding is an accumulation of water over dry land, in response to rising water levels by rivers, lakes, and seas. River flooding can come from high snowmelt and heavy rains, with water overflowing banks. Sea level rise increases the probability of flooding during coastal storms and places tens of millions of people at risk worldwide. Climate change has led to heavier precipitation and storm fronts and long‐term sea level changes from melting ice sheets. The oceans have risen approximately 8 inches in just over a century—a rate higher than at any time in the last 2000 years.^63^

Flooding is a widespread hazard that can have serious mental and physical effects on women's health.^64^, ^65^, ^66^ These include pollution and exposure to serious toxic substances, prolonged exposure to waterlogged and unsanitary environments, serious stress, anxiety, and depression, cultural norms that interfere with women's ability to survive floods, and food insecurity. Flooding in Bangladesh, for example, was accompanied by a disproportionate effect on women because of both cultural inhibitions and inadequate social services.^67^ Menstrual problems, urinary infections, pregnancy complications, and malnutrition were observed in women from six villages.^67^ Natural disasters such as hurricanes have been attributed to increased risks for maternal hypertensive disease and significant mood disorders such as depression and post‐traumatic stress; such events have also been shown to increase risk for premature birth, low birthweight, and adverse neurodevelopmental outcomes in offspring such as mood disorders.^12^, ^68^, ^69^ A recent report from the Food and Agriculture Organization shows that increased floods, droughts, and wildfires, as a result of worsening climate change, are having a major deleterious impact on food security, with greatest burden on low‐ and middle‐income countries (LMICs).^70^ Moreover, food insecurity in LMICs due to sociocultural, political, and economic factors is a risk factor for maternal depression and anemia and adverse pregnancy and neonatal outcomes, including pre‐eclampsia, preterm birth, and low birthweight.^71^ It is anticipated that floods, droughts, and wildfires will further exacerbate food insecurity for women, especially in LMICs, and result in even greater risks for adverse pregnancy and neonatal outcomes in the future if the climate crisis is not abated.

### 3.5 Vector‐borne illnesses

More than 1 million people die every year from vector‐borne illnesses such as malaria, dengue, schistosomiasis, congenital Zika, and Chagas disease.^72^, ^73^ Both temperature and precipitation impact the survival and development of these vectors. Indirect effects of climate change relate to pathogen exposures that develop in response to changes in temperature, fluctuations in water levels, and redistributions of normal or newly emergent species. Malaria kills 1 million people every year, and pregnant women are especially vulnerable. Malaria infection during pregnancy can have adverse effects on both mother and fetus, including maternal and fetal anemia, miscarriage, intrauterine demise, preterm delivery, intrauterine growth restriction, low birthweight infants, and maternal and/or neonatal death.^74^ Zika virus carries a unique risk in pregnancy and has been associated with birth defects, most notably severe microcephaly.^75^ A recent report modeling environmental alterations expected with climate change and socioeconomic scenarios predicts that 1.3 billion new people could face transmission temperatures for Zika virus by 2050, with increased risk including North America and Europe.^76^ A rise in temperature will also increase transmission by increasing the range of mosquitoes that transmit malaria. Previously stable distributions of vectors have been shifting in response to climate change.^77^ Thus, decreasing the global incidence of vector‐borne illnesses is essential for maternal and neonatal health globally.

Although not a vector per se, air pollution has been associated with increasing the spread and severity of COVID‐19 and other respiratory infections.^78^ In recent years, wildfires resulting from ignition of dehydrated vegetation all over the world have exposed millions of people to dangerous levels of PM_2.5_. This exposure in turn has been associated with a significantly higher mortality rate from severe acute respiratory syndrome coronavirus 2 (SARS‐CoV‐2) infection—estimated to be as much as 77% higher with wildfire smoke exposure than without.^79^

Thus, as the global climate crisis leads to numerous environmental catastrophes, these in turn interact, resulting in a cascading series of harms to humans and especially the most vulnerable.

## 4 SOCIAL JUSTICE

### 4.1 Sexual and reproductive health and rights

A major consequential effect of climate change on human populations is the migration and disruption of mass populations in response to adverse conditions. Heat and drought disrupt agriculture and thus quantity and quality of food and water, with resultant mass migration as populations try to find better conditions for survival. As noted above, global temperature extremes, droughts, floods, and impacts on food and water disproportionately affect the health of women who are more vulnerable to these effects due to their social status and family responsibilities. Women and girls are at higher risk of sexual violence, sexual exploitation, abuse, trafficking, and intimate partner violence in response to displacement and their vulnerability. Psychological stress, anxiety, and depression are all common. The link between climate change and sexual and reproductive health and rights was recently summarized in a review by Women Deliver.^11^ Therein, climate change is deemed “not gender neutral,” as it increases social inequities, with “gender, sexuality, age, wealth, indigeneity, and race all determining factors in the vulnerability to climate change”.^11^ Another document from this group underscores the need to prioritize and incorporate sexual and reproductive health and rights in national action plans and governmental policies for climate change mitigation and strategies long term.^80^ A case study of such an approach is presented in Section 6.

### 4.2 Environmental injustice

Environmental injustice—the disproportionate exposure of communities of color and the poor to pollution, and resulting exacerbation of health inequities due to unequal environmental protection through laws, regulations, governmental programs, enforcement, and policies^81^—is exacerbated by climate change. Thus, the effects of climate change that disproportionally affect disadvantaged communities (including those of color and low income) and lower‐income countries, and their impacts on environmental justice, must be also considered and addressed.^81^, ^82^, ^83^, ^84^ A recent report by the US Environmental Protection Agency concluded that Black and African American, Hispanic and Latino, American Indian and Alaska Natives, and Asian individuals are most likely to live in an area with the highest impacts of climate change.^85^ Not only do socioeconomic and structural disparities exacerbate the impact of environmental exposures and climate change‐related impacts that are experienced by individuals and populations, they are rendered further unjust by the unequal contributions of high‐ and low‐resource populations to the carbon load of the atmosphere.^86^

### 4.3 Intergenerational injustice

It is now clear that the ongoing climate crisis poses significant risks to women, pregnant mothers, unborn fetuses, and offspring who were exposed in utero to climate stressors. Thus, adverse effects will reverberate across the human lifespan, with individuals born disadvantaged from in utero climate‐related insults, thereby burdened with predispositions to disease (e.g. obesity, metabolic disorders, congenital defects, allergies, and neurodevelopmental and psychological impairments) and ill‐adapted to further climate insults during their own lifetimes.^1^, ^3^, ^87^

As the earth is warming and as portions of the planet become uninhabitable in the coming decades at the current rate of anthropogenic emissions, climate change now poses an existential threat to a great portion of humanity. Women's healthcare providers have a significant and beneficial role to play in assisting their patients with adaptation to a changing climate; these efforts will have long‐term impacts and can soften the blow to future generations.

## 5 CLIMATE CHANGE AND TOXIC CHEMICALS

Climate change and environmental chemical toxicants are interactive in some of their effects.^88^ Warmer temperatures increase exposure to toxic chemicals and the impacts of extreme weather events include concentrated releases of chemicals^89^; climate change can exacerbate the health impacts of air pollution, and toxic chemicals may hinder the physiologic adaptation to climate change and increase the vulnerability of communities, especially in LMICs, to climate change effects.^89^ Fossil fuel use is a primary driver of climate change, accounting for almost two‐thirds of the contribution to greenhouse gas emissions.^90^, ^91^ Fossil fuels, including petroleum and natural gas, are the ingredients for the production of petrochemicals, which are in turn used in consumer products, including plastics.^92^ These chemicals include phthalates, flame retardant chemicals, and perfluorinated compounds, which are all endocrine‐disrupting chemicals and can increase the risk of adverse health effects. These chemicals are measured ubiquitously in sediments globally and in humans.^93^, ^94^ The production of these chemicals, which has steadily increased since the 1950s, corresponds to a dramatic increase in the incidence of health outcomes that are impacting women and their families (i.e. obesity, diabetes, fertility problems, cancer, and neurodevelopmental disorders).^95^ Thus, the sources of climate change are also linked to toxic chemical pollution.

## 6 A CASE STUDY: CLIMATE CHANGE, DISASTER PLANNING, AND THE PHILIPPINES

While addressing the root causes of climate change, health professionals, governmental leaders, the private sector, and civil society must prepare for the consequences of climate change through disaster planning and risk reduction, education, and advocacy.

Climate‐induced extreme weather events are becoming more frequent and more intense. The Philippines is a disaster‐vulnerable country accustomed to many natural and man‐made disasters due to its geographic location in the typhoon belt and the Pacific Ring of Fire. Several natural hazards such as cyclones, floods, sea level rise, droughts, earthquakes, volcanic eruptions, and landslides are almost a way of life for people in its many regions. The 2020 World Risk Index ranked the Philippines ninth out of 173 countries in terms of disaster risk.^96^ While the ranking improved from second place in 2014 to third in 2015 to 2018, the Philippines maintained its rank from 2019 to 2020. It has remained highly exposed to the effects of natural hazards.^97^ According to the National Disaster Risk Reduction and Management Council (NDRRMC), eight of the 10 most expensive tropical storms occurred in the last decade in the Philippines. The NDRRMC, created in 2010 under Republic Act 10121, performs policymaking, coordination, integration, and supervisory functions in the monitoring of preparation, implementation, and evaluation of the National Disaster Plan.^98^

Over the years, the Philippines has adopted various approaches to managing disasters. From the 1970s to the current state, disaster planning and management approach have evolved from disaster preparedness and response, disaster risk management, and eventually disaster risk reduction. A “whole of society approach,” involving many key government sectors and agencies including civil society and private business, underscores the need for multisectoral environmental risk management. Prevention of deforestation to increase livelihood and consumption leading to environmental degradation will potentially cause societal fragmentation. This anti‐environment activity has negative consequences that can ultimately impact the health and well‐being of people.^99^

The Department of Health operates under the Health Cluster of the NDRRMC and ensures the protection, safety, and welfare of the people in times of disaster.^100^ To date, the government has been investing in raising awareness, developing geospatial data and mapping tools to assist in disaster responses, upskilling of staff, and procurement of necessary equipment to upgrade agencies involved in climate change and disasters. There has been heightened public consciousness and increased engagement of key stakeholders since Typhoon Haiyan in 2013 and the ongoing COVID‐19 pandemic. There is an initiative to increase access to and availability of the internet and social media in promoting information and education. Current literature indicates some evidence of why the country is vulnerable. Mapping of the effects of climate change to 11 offshore zones and specific risks such as sea level rise, extreme rainfall events, extreme heating events, increased ocean temperatures, and a problematic water budget has been pursued.^101^, ^102^

The climate‐related health effects of typhoons and floods on malnutrition, mainly undernutrition, have been demonstrated in households and individuals and are associated with risk factors such as household and maternal characteristics on a background of extreme weather events, needing pre‐disaster health preventative strategies.^103^, ^104^ Many of the possible factors act indirectly or intersect with other determinants in different levels and bring about poor Filipino health outcomes. As a signatory to the Paris Agreement on Climate Change in 2017, the Philippine government must accelerate mitigation and adaptation strategies amidst worsening existing inequalities and vulnerabilities. The National Climate Change Action Plan 2011–2028 and the Department of Environment and Natural Resources Strategic Program on Climate Resilience for 2018–2022 has been working to mainstream climate change and disaster risk reductions, particularly in vulnerable provinces and major urban centers like Metro Manila, Cebu, Iloilo, and Davao.^105^, ^106^

The Philippines, with its nearly 110 million population, has demonstrated that vulnerability to physical hazards can intersect with social determinants and produce disasters. Over the last few years since Typhoon Haiyan, the country is learning that wider cooperation of multiple sectors and strengthening a fragile health system are essential. Individual and collective action and the initial gains of building adaptive and resilient communities as more awareness and information are cascaded to the citizens need to be sustained. The next challenge is a biological disaster, the COVID‐19 pandemic, which threatens the way of life of everyone. Health professional organizations can be in the forefront of environmental health advocacy to save the present generation and future generations through engagement as public opinion leaders.^107^ This story is still unfolding.

## 7 CONCLUSION

The ongoing climate crisis poses significant risks to women, pregnant mothers, unborn fetuses, and offspring who were exposed in utero to climate stressors, especially those in marginalized communities where effects are magnified. Thus, adverse effects will reverberate across the human lifespan, with individuals born disadvantaged from in utero climate insults, burdened with predispositions to disease (e.g. obesity, metabolic disorders, congenital defects, allergies, neurodevelopmental and psychological impairments, etc), and ill‐adapted to further climate insults during their own lifetimes.^87^, ^108^

It is also clear that the health of women is affected disproportionately due to the climate crisis, although they contribute little to its genesis. In protecting pregnant women and the developing fetus from the health hazards associated with air pollution, heat, and natural disasters, any individual efforts—while commendable—are insufficient and beyond individual control due to the systematic sources of the climate change problem, of which the fossil fuel industry is an important contributor. Pregnant women already face a litany of personal restrictions from dietary limitations, travel precautions, and personal care product choices. They cannot control the outdoor air quality they encounter or the ambient local temperature. Thus, we recommend climate policy interventions that address sexual and reproductive health and human rights as key to improving the lives of women and girls around the globe. In particular, we join with governments and authoritative bodies that have identified policies that reduce fossil fuels as key to addressing climate change.^109^ Overexploitation of fossil fuels has been linked to the catastrophic climate events we are now experiencing. Efforts to reduce the contribution of fossil fuel production to climate change must also simultaneously ensure these solutions also focus on reducing toxic chemical production via fossil fuel contributions.

**FIGURE 2:**
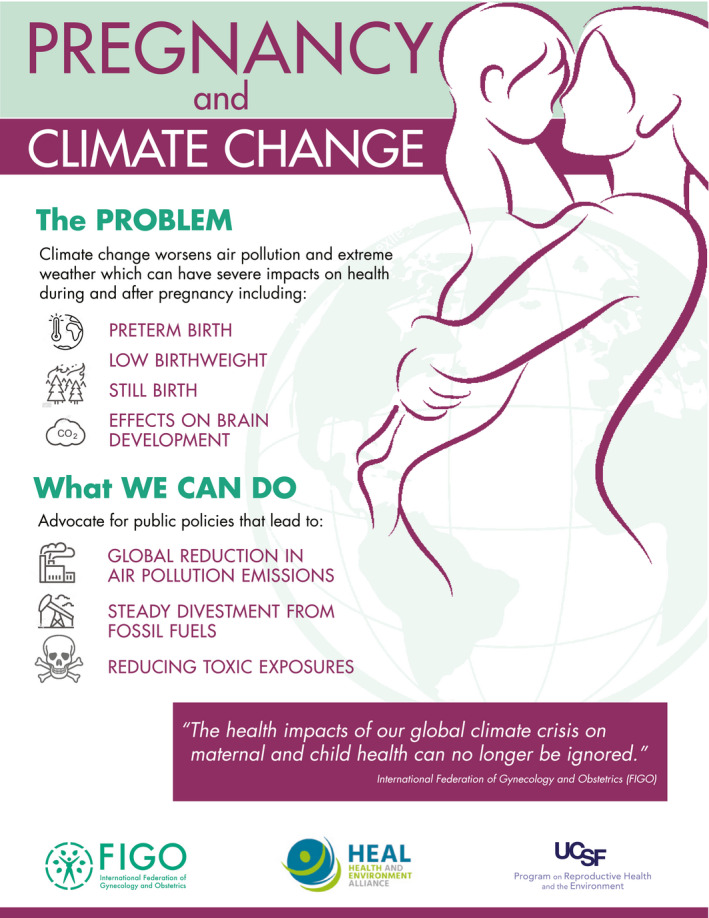
Infographic for health advocates and policymakers, giving an overview of the ways in which climate change increases health risks to pregnant women and their children. Available in nine languages: English, German, French, Spanish, Portuguese, Italian, Polish, Bosnian and Serbian https://www.env‐health.org/climate‐change‐puts‐pregnant‐women‐at‐greater‐risk‐new‐infographic‐by‐figo‐ucsf‐and‐heal/, Accessed September 27, 2021

As the earth is warming and as portions of the planet become uninhabitable in the coming decades at the current unabated rate of anthropogenic emissions, climate change now poses an existential threat to a great portion of humanity. Women's healthcare providers have a significant and beneficial role to play in assisting their patients with adaptation to a changing climate; these efforts will have long‐term impacts and can soften the blow to future generations. OBGYNs throughout the world can educate their patients about the health harms of climate change and advocate for policies promoting reduced sources of climate change pollutants, including the contributions from the fossil fuel industry, carbon neutrality, and environmental justice (see Figure 2). Furthermore, clinicians should consider the carbon footprint of the care they provide; indeed, the healthcare industry is responsible for 1%–5% of total global environmental impacts, and in some nations health care accredits >5% of global health impacts.^110^

We recommend that the Climate Crisis be recognized for the global emergency that it is,^1^, ^2^, ^3^, ^4^ and that healthcare providers lead in the pillars that FIGO has identified: advocacy, research interpretation, capacity building, and education. A focus on education, research, and advocacy in responding to changing health consequences and global awareness are key to educating our professional healthcare providers, patients, the lay public, and ministers of health and other leaders, and making the changes necessary to address this crisis. In 2021, editors of 220 leading medical, nursing, and public health journals worldwide, in an unprecedented joint statement, called the rapidly warming climate the “greatest threat” to global public health and urged world leaders to urgently cut heat‐trapping emissions to avoid “catastrophic harm that will be impossible to reverse”.^111^ FIGO’s journal, the *International Journal of Gynecology and Obstetrics*, was a co‐signatory of this statement. FIGO is incorporating climate change into its education, advocacy, and research programs within its Committee on Climate Change and Toxic Environmental Exposures so that global leaders from our member organizations have the ability to effect change in their countries, regions, and globally.

## CONFLICTS OF INTEREST

The authors have no conflicts of interest.

## AUTHOR CONTRIBUTIONS

The topic for this article derived from discussion within the FIGO Committee on Climate Change and Toxic Environmental Exposures. JC wrote the first draft with LG. Other authors wrote sections and all authors reviewed multiple drafts of the manuscript and gave further input. LG incorporated and edited all comments and adjudicated the text and references to journal style. All authors contributed intellectually to all aspects of this manuscript, and the manuscript was discussed at the quarterly meetings of the FIGO Committee on Climate Change and Toxic Environmental Exposures, and the near‐final draft was subsequently reviewed by, and consensus derived from, all Committee members.
